# Metabolism and Yield of Grape–Tomato Hybrids Under Heat Stress in an Innovative Protected Environment Using Twin-Walled Polycarbonates with Laminar Water Flow

**DOI:** 10.3390/metabo16060389

**Published:** 2026-06-04

**Authors:** Robert Ramsay Garcia, Aline Nunes, José Advan Pereira Pedrosa Junior, Renê Arnoux da Silva Campos, Franciely da Silva Ponce, Joangela Oliveira de Moura Ramsay, Márcio Roggia Zanuzo, Silvia Graciele Hulse de Souza, Flávio Fernandes Junior, Sílvia de Carvalho Campos Botelho, Santino Seabra Junior

**Affiliations:** 1 Mato Grosso State University (UNEMAT), Cáceres 78200-000, Mato Grosso, Brazil; ramsay.robert@unemat.br (R.R.G.); junior.jose@unemat.br (J.A.P.P.J.); renecampos@unemat.br (R.A.d.S.C.); joangela.moura@gmail.com (J.O.d.M.R.); 2 Institute of Biosciences, São Paulo State University (UNESP), Botucatu 18618-689, São Paulo, Brazil; nunes1@unesp.br; 3 School of Agricultural Sciences, São Paulo State University (UNESP), Botucatu 18600-950, São Paulo, Brazil; franciely.ponce@unesp.br; 4 Federal University of Mato Grosso (UFMT), Sinop 78550-728, Mato Grosso, Brazil; marcio.zanuzo@ufmt.br; 5 Federal University of Paraná (UFPR), Jandaia do Sul 86900-088, Paraná, Brazil; silviahulse@ufpr.br; 6 Brazilian Agricultural Research Corporation (EMBRAPA Vegetables), Brasília 72305-970, Brazil; 7 Brazilian Agricultural Research Corporation (EMBRAPA Agrosilvopastoral), Sinop 78550-970, Mato Grosso, Brazil; silvia.campos@embrapa.br

**Keywords:** *Solanum lycopersicum* L., microclimate regulation, evaporative cooling, carotenoid biosynthesis

## Abstract

**Background/Objectives**: The high temperatures associated with climate change represent an important constraint for tomato production in tropical regions, affecting plant growth, reproductive development, and fruit metabolic composition. In this context, protected cultivation systems capable of modifying greenhouse microclimates may help reduce thermal stress and maintain crop productivity. **Methods**: This study evaluated the effects of two protective environments, diffuse agricultural film (AF) and twin-walled polycarbonate panels with laminar water flow (P), on the agronomic performance and fruit metabolic traits of five grape–tomato hybrids grown under tropical conditions. Microclimatic variables, vegetative growth, yield components, postharvest behavior, and fruit quality attributes were evaluated, with emphasis on carotenoid accumulation. **Results**: Compared with the agricultural film environment, the polycarbonate system reduced global radiation and photosynthetically active radiation (PAR) and was associated with an increase in yield of approximately 25%, an increase in fruit number of approximately 13%, and an 8% increase in fruit diameter. In addition, some hybrids cultivated under the polycarbonate system showed greater lycopene and β-carotene accumulation, indicating that microclimate moderation may favor carotenoid-related fruit quality depending on genotype. Principal component analysis revealed a clear separation between cultivation environments, with the polycarbonate system more closely associated with yield-related and canopy development traits, whereas the agricultural film environment was linked to biomass accumulation and selected physicochemical attributes. Among the evaluated hybrids, BS IGR0104, Jacy, and GI7545 showed the most favorable combination of agronomic performance and fruit quality traits. **Conclusions**: These results demonstrate the importance of climate-adaptive protected cultivation systems and hybrid selection for improving tomato productivity under tropical heat conditions.

## 1. Introduction

The rising temperatures associated with climate change have become a major constraint to horticultural production, particularly in tropical and subtropical regions where thermal conditions frequently exceed the optimum range for crop growth. In tomato (*Solanum lycopersicum* L.), heat stress negatively affects photosynthesis, carbon assimilation, pollen viability, fruit set, and ultimately fruit yield and quality [1,2,3,4]. These limitations are particularly relevant for grape–tomato hybrids, whose commercial value depends on both yield stability and on maintaining fruit quality and bioactive compounds under stressful environments.

Protected cultivation systems have been increasingly adopted to reduce environmental constraints and stabilize vegetable production throughout the year [5]. By partially regulating the internal microclimate, these systems can improve water use, reduce biotic pressure, and enhance crop performance [6]. However, under warm climate conditions, conventional greenhouse structures may also intensify heat accumulation, exposing plants to supraoptimal temperatures and compromising physiological performance [7]. Therefore, improving thermal regulation inside protected environments remains a central challenge for sustainable horticultural production in tropical regions [8,9].

The internal microclimate of protected environments is strongly determined by the covering material [10,11]. In Brazil and other tropical regions, polyethylene films are widely used because of their low cost and availability; however, their limited thermal insulation often results in excessive heat buildup [5,10,12]. In this context, polycarbonate coverings have emerged as a promising alternative due to their favorable optical and physical properties, including durability, mechanical resistance, high light transmittance, and lower thermal conductivity [13,14,15,16]. Twin-walled polycarbonate panels are particularly attractive because they improve light diffusion and may promote a more uniform distribution of photosynthetically active radiation (PAR) inside the canopy [16,17].

Recent technological advances have expanded the potential of polycarbonate-based structures through the integration of cooling strategies designed to mitigate heat stress [16,18]. One such approach is the use of laminar water flow through the internal channels of twin-walled polycarbonate panels. This system is expected to reduce heat load, attenuate thermal fluctuations, and improve greenhouse microclimate without the high energy demand commonly associated with active cooling systems [14,19]. As such, it may represent an efficient alternative for protected cultivation under high-radiation environments.

From a physiological and metabolic perspective, heat stress causes substantial alterations in plant functioning, including reductions in photosynthetic efficiency, increases in respiratory demand, and disturbances in redox homeostasis [20]. These effects directly influence the synthesis and accumulation of important fruit metabolites such as sugars, organic acids, carotenoids, and phenolic compounds, which determine fruit quality, nutritional value, and marketability [21,22].

Among tomato fruit metabolites, lycopene and β-carotene are particularly important because they represent the major carotenoids and fat-soluble antioxidants responsible for fruit nutritional and functional quality [2]. Lycopene is the predominant carotenoid associated with the characteristic red coloration of ripe tomato fruits and is recognized for its strong antioxidant capacity, especially in scavenging singlet oxygen species [6]. In contrast, β-carotene contributes to antioxidant protection while also serving as an important precursor of vitamin A in the human diet. Because carotenoid biosynthesis is highly sensitive to environmental conditions, particularly temperature and radiation, these compounds represent important metabolic indicators of fruit quality under heat-stress conditions [2,3]. In tomato, elevated temperatures can impair lycopene biosynthesis, alter carbon partitioning, and reduce antioxidant potential, thereby compromising both yield and functional quality [23,24].

Although heat stress impacts on tomato production have been widely studied, few investigations simultaneously evaluated microclimate, yield formation, and fruit metabolic quality while using innovative greenhouse cooling technologies. In particular, the combined effects of twin-walled polycarbonate coverings and laminar water cooling on grape–tomato hybrids grown under tropical heat stress are still poorly understood. This gap limits the development of climate-resilient greenhouse systems that can improve both agronomic performance and fruit quality under increasingly unfavorable thermal conditions.

Therefore, this study aimed to evaluate how an innovative greenhouse system equipped with twin-walled polycarbonate panels and laminar water flow can affect the microclimate, yield performance, and fruit metabolic traits of grape–tomato hybrids grown under heat-stress conditions. This study aims to provide new insights into the potential of advanced protected cultivation systems to mitigate thermal stress and enhance tomato productivity and quality in warm-climate regions.

## 2. Materials and Methods

### 2.1. Experimental Site and Growing Conditions

The experiment was conducted in Sinop, Mato Grosso State, Brazil (11°51′42.6″ S, 55°36′45.1″ W; 370 m altitude), a region classified as Aw according to the Köppen climate classification, characterized by a tropical climate with a rainy summer and dry winter. The mean annual temperature is 25.4 °C, and the average annual rainfall is approximately 1801 mm. The study was carried out in two protected cultivation environments designed to provide contrasting thermal conditions under controlled management.

### 2.2. Experimental Design and Plant Material

The experimental design was a randomized complete block design arranged in a 2 × 5 factorial scheme, consisting of two cultivation environments: diffuse agricultural film (AF) and alveolar polycarbonate panels with laminar water flow (P), and five grape–tomato (*S. lycopersicum* L.) hybrids: ‘BS IGR0104’, ‘Guaraci’, ‘Jacy’, ‘GI7545’, and ‘BS IGR0048’. Seeds were obtained from BlueSeeds^®^ (Holambra, São Paulo, Brazil). All hybrids were characterized by an indeterminate growth habit and commercial relevance. Each treatment consisted of four replicates, and each experimental plot comprised eight pots with one plant per pot. Evaluations were performed using the five central pots to avoid border effects. Plants were spaced 0.50 m apart within rows, with 1.20 m between rows.

Prior to transplanting, soil acidity correction and base fertilization were performed. Dolomitic limestone was applied to adjust soil pH, followed by the incorporation of 40 t ha^−1^ of well-composted cattle manure and 3400 kg ha^−1^ of single superphosphate (18% P_2_O_5_), applied 30 days before the establishment of the experiment. Nutrient supply during crop development was provided through fertigation, following technical recommendations for tomato cultivation. The topdressing fertilization consisted of weekly applications of calcium nitrate (120 g plant^−1^; 15% N and 19% Ca), potassium sulfate (40 g plant^−1^; 48% K_2_O and 15% SO_4_), monoammonium phosphate (30 g plant^−1^; 12% N and 61% P_2_O_5_), potassium nitrate (110 g plant^−1^; 13% N, 44% K_2_O, and 1.5% S), and magnesium sulfate (70 g plant^−1^; 9% Mg and 12% S). Fertilizers were supplied at seven-day intervals through the fertigation system, following the guidelines proposed by Stoleru et al. [25].

Irrigation management was based on crop water demand, with an average daily application of 3.5 mm, adjusted to compensate for evapotranspiration losses estimated for each cultivation environment, according to Gong et al. [26]. Irrigation was uniformly applied in both environments to avoid water availability being a confounding factor. Pest and disease management followed standard commercial recommendations for tomato cultivation, with preventive and corrective measures applied whenever necessary to maintain plant health and avoid interference with experimental treatments.

### 2.3. Greenhouse Structure and Cooling System

Both experimental units consisted of gable-roof greenhouses measuring 6.40 m × 20.00 m, with a sidewall height of 3.5 m and a central height of 4.8 m. The sidewalls were covered with 30% shading Aluminet^®^ (Ginegar Polysack, Ma’ale Gilboa, Israel) reflective screens. Both structures were oriented in the north–south direction.

One greenhouse was covered with a 150 μm diffused polyethylene film with UV protection (88% light transmission). The second greenhouse was covered with transparent 10 mm twin-walled polycarbonate panels (Polisystem^®^, Polisystem S.r.l., Abruzzo, Italy).

In the polycarbonate-covered structure (P), water continuously circulated through the internal alveolar channels of the twin-wall panels, forming a thin laminar film along the internal surfaces (Figure S1). The system operated using a recirculating 1000 L reservoir connected to an automated pumping system programmed to cycle on for 30 min and off for 10 min between 06:00 and 18:00 h. Water circulation aimed to reduce heat load and attenuate radiation peaks by partially absorbing infrared energy before it entered the greenhouse environment. This process contributed to greater thermal stability and moderated microclimatic fluctuations inside the protected cultivation system.

### 2.4. Seedling Production and Crop Management

Seedlings were produced in 128-cell expanded polystyrene trays filled with commercial substrate (Carolina^®^, Santa Cruz do Sul, Rio Grande do Sul, Brazil) and maintained under protected conditions. Sowing was performed on 22 April 2023, and transplanting was carried out 30 days after emergence.

Plants were cultivated in 5 L pots containing a 1:1 (*v*/*v*) mixture of commercial substrate (Carolina^®^, Class LXXVI) and carbonized rice husk. The commercial substrate consisted of 70% sphagnum peat and 30% expanded vermiculite, supplemented with dolomitic limestone, agricultural gypsum, and NPK fertilizer. According to the manufacturer’s specifications, the substrate presented a pH of 5.5 ± 0.5, electrical conductivity (EC) of 0.7 ± 0.3 dS m^−1^, bulk density of 145 kg m^−3^, and water-retention capacity of 55%.

### 2.5. Nutrient Solution, Irrigation Management and Environmental Monitoring

The nutrient solution was prepared according to the formulation proposed by Furlani et al. [27]. Nutrient solution management began immediately after transplanting. Irrigation events were scheduled according to crop developmental stages and adjusted to maintain adequate substrate moisture throughout the cultivation cycle. The irrigation volume was defined to ensure uniform wetting of the substrate and to promote a drainage fraction of approximately 10–20%, preventing salt accumulation in the root zone.

After transplanting, nutrient solution was supplied at 50% of the recommended concentration and increased to 100% during the vegetative stage, maintaining electrical conductivity (EC) between 0.60 and 0.80 dS m^−1^. Plants received 0.375 L per irrigation event in five daily irrigations, totaling 2.25 L plant^−1^ day^−1^.

During the emergence of the first flower buds and fruits, nutrient solution was applied at full strength, maintaining EC between 0.80 and 1.10 dS m^−1^, with 0.375 L supplied in eight daily irrigations (3.00 L plant^−1^ day^−1^). At the initial fruit development stage, nutrient solution was applied at 80% of the recommended concentration, maintaining EC between 0.90 and 1.40 dS m^−1^, with 0.675 L per irrigation event distributed across ten daily irrigations (6.75 L plant^−1^ day^−1^). A 1000 L reservoir supplied the nutrient solution to both experimental environments, supporting a total of 280 plants.

Microclimatic conditions were continuously monitored in each cultivation environment using an automated weather station (ONSET HOBO U30, Bourne, MA, USA). Sensors were installed at canopy height and recorded the following variables: global solar radiation (W m^−2^), photosynthetically active radiation (PAR, µmol m^−2^ s^−1^), air temperature (°C), and relative humidity (%). Measurements were recorded at 15 min intervals throughout the experimental period, from July to October 2023.

To provide reference environmental conditions, an additional weather station (Campbell Scientific Inc., Logan, UT, USA) was installed outside the protected environment. Daily averages and diurnal variation patterns were calculated to compare the microclimatic behavior of the cultivation systems.

### 2.6. Vegetative Growth and Yield Evaluation

Vegetative growth traits were evaluated at the end of the harvest period for each hybrid in both cultivation environments. Each hybrid plot was considered one experimental unit, and five plants were randomly selected from the central area of each plot to avoid border effects. Plant height (cm) was measured using a measuring tape from the base of the stem at the substrate surface to the apical meristem. After, plants were then separated into leaves and stems. Leaf area was determined using a LI-3100C Area Meter (LI-COR Biosciences, Lincoln, NE, USA). Plant samples were labeled and dried in a forced-air oven (Solab SL-102) at 55 °C until constant weight (approximately 15 days). After drying, samples were weighed using a precision electronic balance to determine dry biomass (g).

Ripe fruits were harvested at maturity stage 6, defined by an intense red color covering more than 90% of the fruit surface. Harvesting was carried out weekly from 20 July to 20 October 2023. For yield evaluation, fruits from all plants within each experimental plot were collected, counted, and weighed using a precision digital scale (Electronic SF-400, Bonvo, Wenzhou, China). Fruit length and diameter were measured using a digital caliper. The following variables were determined: fruit length (mm), fruit diameter (mm), mean fruit mass (g fruit^−1^), number of clusters per plant number of fruits per plant and total yield (kg plant^−1^).

### 2.7. Sample Preparation

For physicochemical and biochemical analyses, six fruits were randomly collected from each experimental plot at each harvest and pooled to form a composite sample representative of that plot.

Fruits were selected according to commercial quality standards, ensuring the absence of mechanical damage, pests, or diseases. After selection, fruits were sanitized by immersion in a sodium hypochlorite solution (100 mL L^−1^) for 10 min, followed by rinsing with distilled water.

Samples were homogenized using a Philco Juicer, and aliquots were transferred into 15-mL Falcon tubes in triplicate (*n* = 3). The samples were properly labeled and stored at −80 °C until physicochemical and biochemical analyses were performed.

### 2.8. Physicochemical Analyses

For colorimetric analysis, fresh fruits were used. Fruit color parameters, including chroma (C*) and hue angle (h°), were determined using a chromameter (Konica Minolta Inc., Tokyo, Japan). Measurements were taken at two equidistant points on the fruit surface.

The pH of the homogenized fruit pulp was measured using a bench pH meter (Incoterm PHB-550, Porto Alegre, Brazil).

The titratable acidity (TA) was determined by homogenizing 20 g of tomato pulp in 100 mL of distilled water and titrating the solution with standardized 0.1 N NaOH. The results are expressed as the percentage of citric acid.

The total soluble solids (TSS) were determined using a digital refractometer (RT-30 ATC, 0–32 °Brix, Reichert Technologies, Depew, NY, USA). The ripeness index was calculated as the ratio between TSS and TA.

### 2.9. Biochemical Analyses

Lycopene and β-carotene contents were determined according to the method described by Nagata and Yamashita [28]. Briefly, 1 mL of tomato pulp was mixed with 4 mL of acetone and 6 mL of hexane and shaken for 20 s. After phase separation, the supernatant was collected and absorbance was measured using a spectrophotometer (Evolution One UV-Vis, Thermo Fisher Scientific, Waltham, MA, USA) at 663, 645, 505, and 453 nm.

Carotenoid concentrations (mg 100 g^−1^ fresh weight) were calculated using Equations (1)–(3):

(1) $$Lycopene=-0.0458A_{663}+0.204A_{645}+0.372A_{505}-0.0806A_{453}$$

(2) $$\beta -Carotene=0.216A_{663}-1.22A_{645}-0.304A_{505}+0.452A_{453}$$

(3) $$Lycopene\;or\;carotene=\frac{\left[\left(A\frac{mg}{100}mL\right)\times mL\;acetone:hexane\right]}{sample\;mass}$$

### 2.10. Postharvest Evaluation

For postharvest shelf-life evaluation, tomatoes were selected and standardized based on uniform size and the absence of mechanical damage, pests, or diseases. Samples consisted of ten fruits per experimental plot. Fruits were stored at room temperature (25 ± 2 °C and 65 ± 5% relative humidity) under laboratory conditions. Fruit mass loss (%) was determined as the percentage difference between the initial fruit mass and the mass recorded at each evaluation time.

Measurements were performed on days 1, 3, 5, 7, 9, 11, 13, and 15 after harvest using an analytical balance (Shimadzu ATX224 Analytical Balance, Kyoto, Japan).

### 2.11. Statistical Analysis

Data were subjected to analysis of variance (ANOVA), and treatment means were compared using Tukey’s test at the 5% significance level (*p* ≤ 0.05) using SISVAR^®^ software (version 5.8). Principal component analysis (PCA) was performed using the singular value decomposition (SVD) algorithm to evaluate relationships among variables across treatments. PCA was conducted using Unscrambler^®^ X software (version 10.4).

## 3. Results and Discussion

### 3.1. Microclimatic Variation Under Protected Cultivation Systems

**Figure 1:**
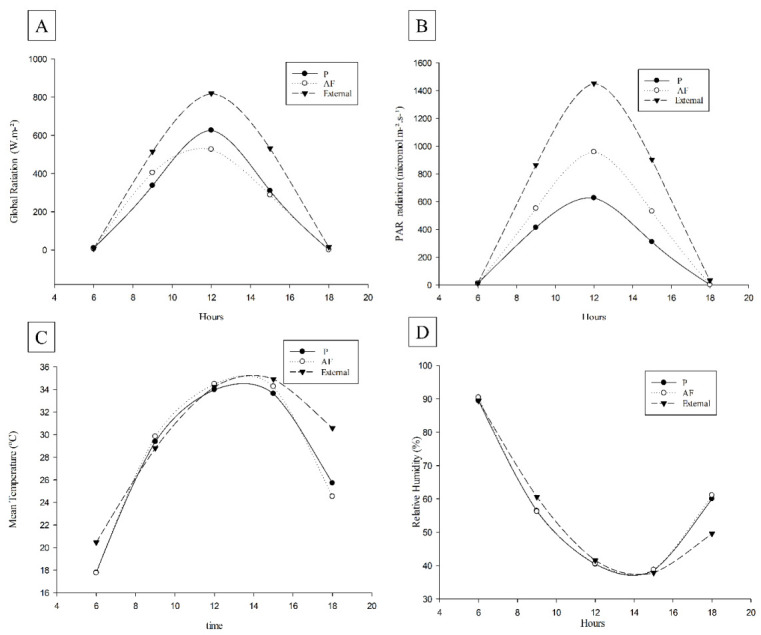
Diurnal dynamics of microclimatic variables across cultivation environments. (**A**) Global solar radiation (W·m^−2^), (**B**) photosynthetically active radiation (PAR, µmol m^−2^·s^−1^), (**C**) air temperature (°C), and (**D**) relative humidity (%). P: twin-walled polycarbonate with laminar water flow; AF: agricultural film; External: open-field conditions.

Protected environments significantly altered the microclimatic conditions compared to the external environment. The diurnal variation of microclimatic variables revealed pronounced differences among cultivation environments, particularly during peak radiation hours (Figure 1), highlighting the effectiveness of protected systems in modulating energy load and atmospheric conditions.

Global solar radiation and photosynthetically active radiation (PAR) followed a typical diurnal pattern, with maximum values observed around midday (Figure 1A,B). However, radiation intensity differed substantially among environments, with the external condition exhibiting the highest values, followed by the twin-walled polycarbonate system with laminar water flow (P), whereas the conventional protected environment (AF) showed the greatest attenuation (Figure 1A). This pattern is consistent with the optical properties of greenhouse covering materials, which influence both the intensity and spatial distribution of incident radiation by enhancing light diffusion and reducing peak irradiance within protected environments [29].

The reduction in PAR in the P environment (Figure 1B) limited the amount of energy effectively available for photosynthetic processes. This reduction is consistent with the optical properties of polycarbonate materials, which promote light diffusion and partial reflectance, thereby decreasing radiation peaks while improving the light distribution within the canopy [29]. Additionally, the laminar water flow likely contributed to further attenuation by absorbing part of the solar spectrum, particularly in the infrared range, reducing the overall energy load entering the system and improving internal microclimatic conditions [30].

Notably, PAR attenuation in the P system does not necessarily constrain photosynthesis. Under tropical and subtropical conditions, irradiance often exceeds the photosynthetic saturation threshold, and excess energy can lead to overexcitation of photosystem II (PSII), triggering photoinhibition and the generation of reactive oxygen species (ROS) [31,32]. This effect is further exacerbated under heat stress, which impairs PSII repair and intensifies oxidative damage [33,34]. Therefore, moderating incident radiation may enhance light-use efficiency and contribute to maintaining photosynthetic integrity by preventing excess energy accumulation.

Air temperature exhibited a consistent diurnal pattern across environments, with maximum values occurring between 12:00 and 14:00 h (Figure 1C). The external environment maintained higher temperatures during the afternoon, whereas the protected systems showed a more pronounced thermal decline toward the end of the day, indicating effective thermal buffering and reduced heat exposure. Relative humidity followed an inverse trend, with minimum values between 12:00 and 14:00 h (Figure 1D). The external environment showed lower humidity and greater fluctuations during peak radiation periods, while the protected systems maintained higher and more stable moisture levels, suggesting enhanced atmospheric water retention.

The interaction between temperature and relative humidity directly influences the vapor pressure deficit (VPD), a key regulator of plant water relations and gas exchange [35]. The higher temperature and lower humidity observed externally likely increased VPD, whereas the moderated conditions in AF and P reduced it. This microclimatic regulation is particularly relevant under heat stress, as elevated temperature and VPD can impair plant water status, disrupt stomatal function, and limit photosynthesis [36]. Thus, the buffering effect in AF and P likely promoted a more favorable balance between energy load and water status, supporting greater physiological stability under stress conditions [35,37,38].

### 3.2. Effects of Protected Environment Conditions and Tomato Hybrids on Growth, Yield, and Fruit Quality

The analysis of variance showed that protected environment conditions (PECs) significantly affected most growth- and yield-related variables, including total dry biomass (DB), leaf area (LA), plant height (PH), total shoot dry mass (SDM), total yield (TY), marketable yield (MY), number of fruits (NF), fruit fresh weight (FW), and fruit diameter (FD) (Table 1). In contrast, no significant effects of PEC were observed for fruit length (FL), color parameters (C* and h°), or chemical attributes such as total soluble solids (TSS), titratable acidity (TA), and the SS/TA ratio.

Tomato hybrids (THs) exerted a broader influence, significantly affecting nearly all evaluated variables, particularly fruit fresh weight (FW), fruit diameter (FD), hue angle (h°), and lycopene and β-carotene contents. However, TH did not significantly influence total yield (Y), marketable yield (MY), or most chemical attributes, except for carotenoids.

The interaction between PEC and TH was significant for several key traits, including DB, LA, PL, SDM, FW, and carotenoid contents, indicating that the response of these variables depends on the specific combination of environmental conditions and hybrid. Conversely, no significant interaction effects were observed for yield components (Y, MY, NF) or most quality-related traits. The coefficients of variation were generally low for most variables, particularly for growth and physical traits.

### 3.3. Vegetative Growth Responses Under Contrasting Protected Environments

The protected cultivation systems generated distinct microclimatic conditions throughout the day (Figure 1), which were reflected in contrasting plant growth responses (Table 2). While the diffuse agricultural film environment (AF) promoted higher total dry biomass (DB) and total shoot dry biomass (SDB) accumulation, the twin-walled polycarbonate system with laminar water flow (P) promoted greater leaf area (LA) expansion (Table 2). These responses suggest that differences in radiation load and atmospheric demand influenced plant growth strategies, biomass production and allocation patterns in grape–tomato hybrid plants.

Higher radiation levels in AF likely enhanced instantaneous carbon assimilation, resulting in greater total biomass accumulation [39]. Tomato biomass production depends on light interception and canopy photosynthesis, and environments with higher irradiance generally promote greater dry matter production when thermal conditions remain within physiological limits [40,41]. For instance, the hybrid GI7545 accumulated the highest biomass under AF, highlighting the positive relationship between radiation availability and biomass production.

In contrast, the moderated microclimate observed in the polycarbonate system favored canopy expansion rather than structural biomass accumulation. The increase in leaf area (LA) under P represented an approximate 35% expansion of canopy surface (Table 2), suggesting a morphological adjustment that allows plants to maintain efficient light interception under reduced irradiance. Increasing leaf area is a well-known strategy to compensate for reduced radiation and sustain canopy photosynthesis in greenhouse crops [42,43,44].

These contrasting responses highlight an important physiological trade-off in tomato cultivation under protected environments. Although plants grown in AF accumulated more total biomass, the microclimate in the polycarbonate system likely reduced thermal and evaporative stress during the hottest hours of the day. Such conditions can improve stomatal regulation and maintain photosynthetic activity, even under lower radiation levels [45]. Previous studies have shown that stable microclimatic conditions can enhance carbon allocation efficiency and improve reproductive performance in greenhouse tomatoes [46,47].

Plant length (PL) showed little variation between environments, suggesting that plant elongation was predominantly genotype-dependent. Among the hybrids, Jacy exhibited the greatest plant height and maintained consistent growth across environments, demonstrating high phenotypic stability under contrasting microclimatic conditions. According to Thakur et al. [48], such stability is a key indicator of genotypic resilience, allowing hybrids to maintain consistent performance across diverse environments.

### 3.4. Yield Under Contrasting Microclimatic Conditions

In this study, the polycarbonate (P) environment significantly enhanced total yield (Y) and marketable yield (MY) across all tomato hybrids compared to the agricultural film (AF) (Table 3). TY increased from 11.5 t ha^−1^ in AF to 14.4 t ha^−1^ in P, representing an approximate 25% increase in productivity. A similar trend was observed for marketable yield (MY), which increased from 2.30 to 2.88 kg plant^−1^ in the P system. This productivity gain was primarily driven by a significant increase in the number of fruits per plant (NF) and mean fruit weight (FW).

Plants cultivated under the polycarbonate (P) system exhibited a 13.3% increase in fruit number (NF), reaching an average of 450 fruits per plant compared to 397 in the agricultural film (AF) (Table 3). Furthermore, the mean fruit weight (FW) rose from 5.9 g in AF to 6.5 g in P, suggesting that the P system enhanced both fruit set and sink strength. While fruit diameter (FD) followed a similar upward trend, increasing from 16.6 mm in AF to 18.0 mm in P, fruit length (FL) remained unaffected by the environment, indicating that longitudinal expansion is controlled by genotypic factors rather than by ambient conditions [49].

The higher yield (Table 3) observed in the twin-walled polycarbonate system with laminar water flow (P) can be directly associated with the microclimatic conditions previously described (Figure 1). The circulation of a thin water layer within the polycarbonate structure reduced peaks of global radiation and photosynthetically active radiation (PAR), moderating the energy load inside the cultivation environment. This attenuation likely prevented excessive radiation and heat stress during the warmest hours of the day, thereby improving physiological efficiency and crop performance. Similar responses have been reported in protected cultivation systems where moderated radiation improves light-use efficiency and maintains photosynthetic stability under high irradiance conditions [50].

Although plants grown under AF accumulated greater biomass due to higher radiation levels (Table 2), this additional energy does not necessarily translate into higher fruit production (Table 3) when plants experience thermal or evaporative stress. In contrast, the moderated microclimate in the P system enhanced canopy development through increased leaf area, likely improving light interception efficiency. A larger canopy surface can help sustain carbon assimilation under moderated radiation levels, ensuring a continuous supply of assimilates for reproductive development [4,41].

In addition, the more stable atmospheric conditions likely optimized the source–sink relationship, favoring the translocation of photoassimilates toward developing fruits. Furthermore, under reduced heat stress, tomato plants are able to maintain reproductive processes more effectively, minimizing flower abortion and improving fruit set [4]. Consequently, these conditions likely supported greater fruit development and fruit number, which was reflected in the increased yield components shown in Table 3.

Genotypic differences among the tomato hybrids were evident for several yield components (Table 3). Although total yield did not differ significantly among hybrids, clear differences were observed in the strategies used to achieve productivity. The hybrid Jacy produced the most fruit per plant, averaging 508.7, indicating a productive strategy focused on greater output. In contrast, GI7545 and BS IGR0104 produced fewer fruits but exhibited greater fruit size, with mean fruit weights close to 7 g per fruit and the largest fruit diameters among the evaluated hybrids. These results suggest a typical trade-off between fruit number and fruit size, a common phenomenon in tomato crops where assimilates are partitioned either into a greater number of fruits or into larger individual fruits [20,51,52].

Among the evaluated hybrids, BS IGR0104, GI7545, and Jacy showed superior productive performance, highlighting the role of genetic background in determining yield components under protected cultivation. To the best of our knowledge, this study represents one of the first reports describing the agronomic performance of these hybrids under protected cultivation conditions, providing useful insights for hybrid selection and management strategies aimed at optimizing tomato productivity under specific microclimatic environments.

### 3.5. Fruit Physical Attributes and Biochemical Composition of Grape–Tomatoes

The physicochemical and biochemical analyses revealed that most fruit quality parameters were relatively stable across cultivation environments, with significant environment–hybrid interactions observed only for lycopene and β-carotene concentrations (Table 1 and Table 4). Among the evaluated variables, statistical differences between environments were detected mainly for hue angle, lycopene, and β-carotene, indicating that pigment-related traits were the most sensitive to microclimatic variation. On average, fruits produced under the agricultural film system (AF) showed a slightly higher hue angle (54.0°), while some hybrids cultivated under the polycarbonate system with laminar water flow (P) showed greater accumulation of lycopene and β-carotene.

The higher hue angle observed under AF suggests a shift toward more yellowish coloration, whereas the lower values recorded under the P environment indicate a deeper red pigmentation associated with lycopene accumulation. According to Borguini and Silva [53], higher hue angle values are typically related to yellow–orange tones, while lower angles indicate a stronger red coloration characteristic of lycopene-rich fruits. This relationship is consistent with the greater carotenoid concentrations detected under the polycarbonate system, suggesting that the moderated microclimate favored pigment biosynthesis [54].

Among the hybrids, GI7545 presented the highest chroma and hue angle values, indicating greater color saturation, whereas Jacy stood out for carotenoid accumulation, reaching 1.40 mg 100 g^−1^ of lycopene. Hybrids BS IGR0104, Guaraci, and Jacy also exhibited relatively high β-carotene concentrations compared with the other genotypes. These results reinforce the strong influence of genetic background on carotenoid accumulation and fruit coloration, which has been widely reported in tomato breeding studies [55,56].

Among the evaluated hybrids, Jacy showed the greatest carotenoid accumulation under the polycarbonate system, reaching 1.9 mg 100 g^−1^ FW and 1.3 mg 100 g^−1^ FW of lycopene and β-carotene, respectively (Table 4). These results reinforce that carotenoid accumulation under moderated microclimatic conditions was genotype-dependent rather than a generalized response across all hybrids.

Carotenoid accumulation in tomato fruits is highly sensitive to environmental factors, particularly temperature and radiation. Lycopene synthesis occurs optimally under moderate temperatures and can be inhibited under excessive heat, which favors the accumulation of carotenoids associated with yellow or orange coloration [6,57]. In the present study, the greater thermal stability observed in the polycarbonate system, characterized by moderated radiation and temperature patterns (Figure 1), likely contributed to the higher carotenoid concentrations detected in specific hybrids (Table 4).

Such conditions may have contributed to maintaining carotenoid biosynthesis under moderated thermal conditions, particularly through the regulation of enzymes involved in carotenoid metabolism, including phytoene synthase (PSY) and lycopene β-cyclase (LCYB) [58]. PSY catalyzes the first committed step of the carotenoid pathway, whereas LCYB catalyzes the conversion of lycopene into β-carotene. In addition to these enzymes, temperature may also affect other enzymes involved in the carotenoid metabolism and degradation pathways [59]. High temperatures may downregulate carotenoid biosynthetic genes while enhancing oxidative degradation and carotenoid cleavage dioxygenase (CCD and NCED) activity, ultimately reducing lycopene accumulation and altering carotenoid profiles under heat-stress conditions [60,61,62].

In contrast, parameters related to fruit taste, including total soluble solids (4.0–4.7 °Brix), titratable acidity (≈0.4%), and the SS/TA ratio, were not significantly affected by the cultivation environment. These values fall within the typical range reported for grape–tomato cultivars and indicate that the microclimatic differences between systems had limited influence on basic flavor attributes. Previous studies have also reported that soluble solids in tomato are influenced primarily by genotype and crop management rather than by moderate variations in protected cultivation environments [63].

Overall, these results demonstrate that while the cultivation environment had limited influence on basic physicochemical traits, it significantly affected pigment accumulation and fruit coloration. The moderated microclimate provided by the twin-walled polycarbonate system with laminar water flow appears to favor carotenoid biosynthesis, enhancing the nutritional and visual quality of grape–tomato fruits.

### 3.6. Post-Harvest Weight Loss Under Contrasting Protected Environments

**Figure 2:**
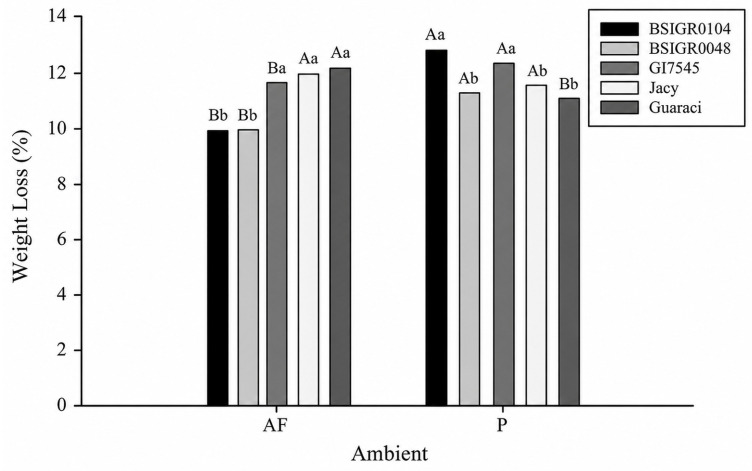
Weight loss (%) of tomato fruits during 15 days of storage at room temperature as affected by protected environment cover (PEC). Fruits were produced under agricultural film (AF) and polycarbonate (P) conditions. Post-harvest evaluations were performed using ten fruits per experimental plot (*n* = 10). Uppercase letters indicate differences between environments within each hybrid, while lowercase letters indicate differences among hybrids within each environment, according to Tukey’s test (*p* ≤ 0.05).

Post-harvest weight loss of tomato fruits during 15 days of storage at room temperature showed significant interaction between cultivation environment and hybrid, although distinct loss patterns were observed over the storage period (Figure 2). Overall, fruits produced under the polycarbonate system (P) tended to maintain better visual quality and slightly lower weight loss compared with those produced under the agricultural film system (AF). BS IGR0048 showed the lowest weight loss under AF (4.71%), while Jacy presented the highest losses under the P environment (12.28%).

Weight loss in tomato fruits during storage is primarily associated with water loss through transpiration and respiration processes, which are strongly influenced by fruit structure, cuticle properties, and pre-harvest environmental conditions. Fruits produced under moderated microclimatic conditions often exhibit improved post-harvest performance due to reduced physiological stress during development [64,65]. The improved microclimate previously observed in the polycarbonate system (Figure 1), characterized by reduced radiation peaks and milder temperatures, may have contributed to better maintenance of fruit quality during storage.

Despite these differences, the magnitude of weight loss observed in this study remained within the range reported for tomato fruits stored at room temperature. Trento et al. [66], for example, reported progressive weight losses in Italian-type tomatoes during storage, reaching approximately 5% after 28 days. The higher values observed in some hybrids in the present study highlight the strong influence of genotype on post-harvest behavior, suggesting that fruit structural traits and water-retention capacity differ among hybrids.

Overall, these results indicate that, while the cultivation environment had a limited direct effect on weight loss, hybrid selection plays an important role in post-harvest performance, with Guaraci and BS IGR0048 showing greater weight retention during storage.

### 3.7. Multivariate Analysis of Morphophysiological and Biochemical Responses Under Contrasting Cultivation Environments

**Figure 3:**
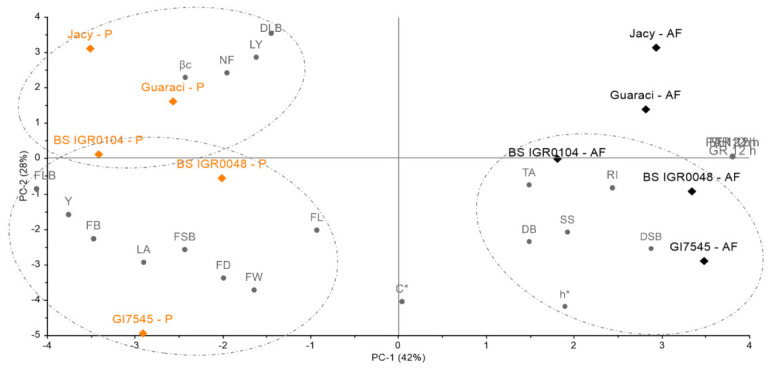
Principal component analysis (PCA) showing the relationships among environmental, morphological, yield, and biochemical variables of tomato hybrids grown under two protected cultivation environments: agricultural film (AF) and polycarbonate with laminar water flow (P). GR (12 h), global radiation; PAR (12 h), photosynthetically active radiation; T (12 h), air temperature; RH (12 h), relative humidity; DB, total dry biomass; LA, leaf area; SDB, shoot dry biomass; Y, yield; NF, number of fruits per plant; FW, fruit weight; FL, fruit length; FD, fruit diameter; C, chroma; h°, hue angle; TSS, total soluble solids; TA, titratable acidity; SS/TA, soluble solids to titratable acidity ratio; RI, ripening index; LY, lycopene; βc, β-carotene.

Principal component analysis (PCA) explained 70% of the total variance, with the first principal component (PC1) accounting for 42% of the variation in the dataset (Figure 3). The ordination clearly separated the two cultivation environments, indicating a strong influence of the protected structure on plant morphophysiological and biochemical responses. Treatments cultivated under the polycarbonate system (P) were predominantly distributed on the left side of the PCA plot, whereas those grown under the agricultural film environment (AF) were positioned mainly on the right quadrants.

In the polycarbonate environment (P), the hybrids Jacy, Guaraci, and BS IGR0104 were closely associated with variables related to reproductive performance and pigment accumulation, including number of fruits per plant (NF), the total shoot dry biomass (SDB), β-carotene (βc), and lycopene (LY). This clustering suggests that the microclimatic conditions provided by the polycarbonate structure favored both vegetative biomass accumulation and carotenoid biosynthesis in tomato fruits. In contrast, the hybrids BS IGR0048 and GI7545 were associated with morphological attributes such as leaf area (LA), fruit diameter (FD), fruit weight (FW), and fruit length (FL), indicating that plant structural growth was strongly expressed for these genotypes under the polycarbonate environment.

In the agricultural film environment (AF), environmental variables such as global radiation, photosynthetically active radiation (PAR), air temperature, and relative humidity were strongly grouped, indicating that plant responses in this system were closely associated with external atmospheric conditions. In this environment, the hybrids BS IGR0104, BS IGR0048, and GI7545 were associated with fruit quality parameters, including titratable acidity (TA), ripening index (RI), total soluble solids (TSS), total dry biomass (DB), shoot dry biomass (SDB), and hue angle (h°). This pattern suggests that the environmental conditions under the agricultural film structure may have influenced metabolic processes related to fruit maturation and biochemical composition.

Overall, the multivariate analysis revealed clear functional differentiation between the two protected cultivation systems. The polycarbonate structure with laminar water flow was primarily associated with variables related to plant growth and fruit carotenoid accumulation, suggesting a more favorable physiological environment for biomass production and pigment synthesis. Conversely, the agricultural film system showed stronger associations with environmental variables and fruit quality attributes related to maturation. These results reinforce the influence of greenhouse microclimate on tomato plant physiological performance, highlighting how moderated radiation and temperature conditions can simultaneously affect plant growth, yield components, and carotenoid accumulation in fruits.

## 4. Conclusions

The twin-walled polycarbonate greenhouse equipped with laminar water flow proved to be the most favorable cultivation environment, as it attenuated radiation peaks and promoted more stable microclimatic conditions compared with the conventional agricultural film system. These moderated environmental conditions improved reproductive performance, resulting in higher fruit number, fruit weight, and overall yield. Among the evaluated hybrids, Jacy, Guaraci, and BS IGR0104 showed the best performance under the polycarbonate environment, which was associated with greater fruit production and higher carotenoid accumulation. In contrast, the agricultural film environment showed stronger associations with fruit physicochemical attributes. Overall, the results demonstrate that the combination of improved greenhouse covering technology and appropriate hybrid selection can enhance the physiological performance and productivity of tomato crops under high-radiation tropical environments. The combination of microclimate regulation and appropriate hybrid selection, therefore, represents a promising strategy to increase the efficiency and sustainability of protected tomato production systems in tropical regions.

## Figures and Tables

**Table 1:** Analysis of variance (F-values) of growth, yield, fruit physical attributes, and biochemical composition of grape–tomato (*Solanum lycopersicum* L.) hybrids, as affected by protected environment conditions (PECs), tomato hybrids (THs), and their interaction.

Variable	PEC	TH	PEC × TH	CV (%)
Growth traits				
Dry biomass (DB)	70.5 *	61.36 *	55.0 *	2.5
Leaf area (LA)	117.1 *	25.15 *	10.25 *	8.4
Plant length (PL)	4.09 *	31.66 *	3.99 *	3.7
Shoot dry biomass (SDB)	94.36 *	22.41 *	7.59 *	2.8
Yield components				
Yield (Y)	13.9 *	1.5 ^ns^	0.0 ^ns^	18.6
Marketable yield (MY)	14.40 *	1.60 ^ns^	0.06 ^ns^	18.43
Number of fruits (NF)	4.7 *	4.3 *	0.3 ^ns^	18.2
Fruit fresh weight (FW)	59.5 *	124.0 *	4.0 *	3.9
Fruit length (FL)	3.0 ^ns^	70.2 *	4.5 ^ns^	3.9
Fruit diameter (FD)	53.7 *	73.8 *	0.9 ^ns^	3.3
Fruit quality traits				
Chroma (C*)	6.3 ^ns^	13.6 *	2.1 ^ns^	6.3
Hue angle (h°)	2.6 ^ns^	50.0 *	3.0 ^ns^	2.6
Total soluble solids (TSS, °Brix)	9.1 ^ns^	2.3 ^ns^	0.4 ^ns^	9.1
Titratable acidity (TA)	14.3 ^ns^	0.2 ^ns^	0.8 ^ns^	14.3
Soluble solids to titratable acidity ratio (SS/TA)	11.2 ^ns^	0.6 ^ns^	0.2 ^ns^	11.2
Lycopene content	7.0 ^ns1^	33.2 *	4.9 *	6.9
β-carotene content	7.9 ^ns1^	6.6 *	3.3 *	7.9

F-values followed by * are significant at *p* < 0.05; ns = not significant (*p* > 0.05). CV = coefficient of variation. ^1^ Lycopene and β-carotene contents were subjected to square root transformation (√y + 1) prior to statistical analysis.

**Table 2:** Effects of protected environment cover (PEC) and tomato hybrid (TH) on total dry biomass (DB), leaf area (LA), plant length (PL), and total shoot dry biomass (SDB) of different hybrids.

PEC	BS IGR0104	Guaraci	Jacy	GI7545	BS IGR0048	Mean (TH)
	DB (g)					
AF	119.2 ± 0.5 ^aB^*	114.9 ± 0.3 ^BC^	111.6 ± 0.5 ^bC^	151.4 ± 0.7 ^aA^	117.2 ± 0.4 ^aBC^	122.8 ^a^
P	114.9 ± 0.3 ^aA^	112.3 ± 0.4 ^aA^	118.3 ± 0.3 ^aA^	116.1 ± 0.5 ^bA^	112.3 ± 0.6 ^bA^	114.8 ^b^
Mean	117.0 ^B^	113.6 ^B^	114.9 ^B^	133.7 ^A^	114.7 ^B^	
	LA (m^2^)					
AF	3.7 ± 0.1 ^bBC^	3.5 ± 0.2 ^bC^	3.5 ± 0.1 ^bC^	4.5 ± 0.4 ^bAB^	4.8 ± 0.2 ^aA^	4.0 ^b^
P	4.9 ± 0.1 ^aB^	4.6 ± 0.2 ^aB^	5.2 ± 0.2 ^aB^	7.2 ± 0.2 ^aA^	5.0 ± 0.5 ^aB^	5.4 ^a^
Mean	4.3 ^BC^	4.0 ^C^	4.4 ^BC^	5.9 ^A^	4.9 ^B^	
	PL (cm)					
AF	38.0 ± 1.8 ^aA^	37.3 ± 1.1 ^aA^	39.0 ± 0.1 ^aA^	32.6 ± 0.6 ^aB^	32.3 ± 1.6 ^bB^	35.8 ^a^
P	38.0 ± 2.2 ^aA^	37.1 ± 1.7 ^aA^	39.0 ± 0.8 ^aA^	32.8 ± 2.1 ^aB^	36.4 ± 1.3 ^aA^	36.7 ^a^
Mean	38.0 ^B^	37.2 ^B^	39.0 ^A^	32.7 ^B^	34.3 ^B^	
	SDB (g)					
AF	87.2 ± 2.1 ^aB^	84.4 ± 3.3 ^aBC^	81.1 ± 2.9 ^aC^	94.0 ± 1.6 ^aA^	88.0 ± 3.3 ^aB^	86.9 ^a^
P	76.3 ± 1.3 ^bC^	75.0 ± 1.6 ^bC^	79.1 ± 2.3 ^aBC^	82.6 ± 2.2 ^bAB^	85.5 ± 2.1 ^aA^	79.7 ^b^
Mean	81.8 ^B^	79.7 ^B^	80.1 ^B^	88.3 ^A^	86.8 ^A^	

* Values are presented as mean ± standard error. Means followed by the same lowercase letters within columns (comparison between protected environment conditions (PECs)) and uppercase letters within rows (comparison among tomato hybrids (THs)) are not significantly different according to Tukey’s test (*p* ≤ 0.05).

**Table 3:** Effects of protected environment cover (PEC) and tomato hybrid (TH) on total yield (Y), marketable yield (MY), number of fruits (NF), fruit weight (FW), fruit length (FL), and fruit diameter (FD) of grape–tomato hybrids.

PEC	BS IGR0104	Guaraci	Jacy	GI7545	BS IGR0048	Mean (TH)
	Y (ton ha^−1^)					
AF	13.3 ± 2.4 ^bA^*	10.4 ± 1.6 ^bA^	10.8 ± 2.5 ^bA^	12.3 ± 3.4 ^bA^	10.9 ± 2.1 ^bA^	11.5 ^b^
P	15.5 ± 1.5 ^aA^	13.5 ± 2.1 ^aA^	14.0 ± 1.6 ^aA^	15.3 ± 2.5 ^aA^	13.6 ± 2.8 ^aA^	14.4 ^a^
Mean	14.4 ^A^	12.0 ^A^	12.4 ^A^	13.8 ^A^	12.2 ^A^	
	MY (kg plant^−1^)					
AF	2.66 ± 0.5 ^aA^	2.08 ± 0.3 ^aA^	2.16 ± 0.5 ^aA^	2.46 ± 0.7 ^aA^	2.17 ± 0.4 ^aA^	2.30 ^b^
P	3.08 ± 0.3 ^aA^	2.70 ± 0.4 ^aA^	2.80 ± 0.3 ^aA^	3.08 ± 0.5 ^aA^	2.71 ± 0.6 ^aA^	2.88 ^a^
Mean	2.87 ^A^	2.39 ^A^	2.48 ^A^	2.77 ^A^	2.44 ^A^	
	NF (units plant^−1^)					
AF	407.5 ± 66.4 ^bAB^	385.0 ± 63.4 ^bAB^	490.2 ± 103.3 ^bA^	372.0 ± 89.2 ^bAB^	330.0 ± 64.0 ^bA^	397.0 ^b^
P	430.7 ± 47.2 ^aA^	492.5 ± 87.3 ^aA^	527.3 ± 75.2 ^aA^	414.5 ± 67.0 ^aA^	386.0 ± 60.5 ^aA^	450.0 ^a^
Mean	419.1 ^AB^	438.7 ^AB^	508.7 ^A^	393.2 ^B^	358.0 ^B^	
	FW (g fruit^−1^)					
AF	6.5 ± 0.1 ^bA^	5.4 ± 0.2 ^bB^	4.4 ± 0.1 ^bC^	6.6 ± 0.4 ^bA^	6.6 ± 0.2 ^bA^	5.9 ^b^
P	7.2 ± 0.1 ^aA^	5.5 ± 0.2 ^aB^	5.3 ± 0.2 ^aB^	7.4 ± 0.2 ^aA^	7.0 ± 0.1 ^aA^	6.5 ^a^
Mean	6.8 ^A^	5.5 ^B^	4.9 ^C^	7.0 ^A^	6.8 ^A^	
	FL (mm fruit^−1^)					
AF	29.6 ± 1.8 ^aA^	22.1 ± 1.1 ^aC^	26.6 ± 0.1 ^aB^	27.1 ± 0.6 ^aB^	28.3 ± 0.5 ^aAB^	26.7 ^a^
P	30.9 ± 1.4 ^aA^	23.0 ± 0.5 ^aC^	24.5 ± 0.2 ^aC^	27.9 ± 1.0 ^aB^	30.3 ± 1.3 ^aA^	27.3 ^a^
Mean	30.3 ^A^	22.5 ^D^	25.5 ^C^	27.5 ^B^	29.3 ^A^	
	FD (mm fruit^−1^)					
AF	18.3 ± 0.8 ^bA^	16.7 ± 0.3 ^bB^	13.7 ± 0.1 ^bC^	18.1 ± 0.1 ^bA^	16.3 ± 0.1 ^bB^	16.6 ^b^
P	19.3 ± 0.8 ^aA^	17.6 ± 0.4 ^aB^	15.4 ± 0.2 ^aC^	19.8 ± 1.1 ^aA^	17.7 ± 1.0 ^aB^	18.0 ^a^
Mean	18.8 ^A^	17.2 ^B^	14.6 ^C^	19.0 ^A^	17.0 ^B^	

* Values are presented as mean ± standard error. Means followed by the same lowercase letters within columns (comparison between protected environment conditions (PECs)) and uppercase letters within rows (comparison among tomato hybrids (THs)) are not significantly different according to Tukey’s test (*p* ≤ 0.05).

**Table 4:** Effects of protected environment cover (PEC) and tomato hybrid (TH) plants on fruit quality attributes of grape–tomato hybrids. Chroma (C*), hue angle (h°), total soluble solids (TSS, °Brix), total acidity (TA, %), soluble solids to acidity ratio (SS/TA), lycopene, and β-carotene.

PEC	BS IGR0104	Guaraci	Jacy	GI7545	BS IGR0048	Mean (TH)
	C*					
AF	36.3 ± 4.7 ^abA^*	36.2 ± 4.4 ^abA^	35.79 ± 4.2 ^bA^	41.0 ± 3.8 ^bA^	38.5 ± 3.6 ^abA^	37.6 ^a^
P	33.9 ± 2.4 ^bA^	34.9 ± 2.2 ^bA^	37.70 ± 3.2 ^bA^	44.6 ± 2.1 ^aA^	38.1 ± 4.0 ^bA^	37.8 ^a^
Mean	35.1 ^B^	35.6 ^B^	36.74 ^B^	42.8 ^A^	38.3 ^B^	
	h°					
AF	54.3 ± 16.6 ^aB^	53.2 ± 15 ^aB^	49.8 ± 11.8 ^aC^	57.3 ± 18.6 ^aA^	55.6 ± 12.0 ^abB^	54.0 ^a^
P	50.3 ± 18.6 ^bC^	49.1 ± 18 ^bCD^	46.3 ± 13.8 ^bD^	57.3 ± 12.5 ^aA^	53.7 ± 19.5 ^bA^	51.3 ^b^
Mean	52.3 ^C^	51.2 ^C^	48.0 ^D^	57.3 ^A^	54.6 ^B^	
	TSS (°Brix)					
AF	4.4 ± 0.3 ^aA^	4.5 ± 0.5 ^aA^	4.7 ± 0.5 ^aA^	4.7 ± 0.2 ^aA^	4.4 ± 0.4 ^aA^	4.5 ^a^
P	4.1 ± 0.3 ^aA^	4.3 ± 0.5 ^aA^	4.3 ± 0.4 ^aA^	4.7 ± 0.6 ^aA^	4.0 ± 0.2 ^aA^	4.3 ^a^
Mean	4.3 ^A^	4.4 ^A^	4.5 ^A^	4.7 ^A^	4.2 ^A^	
	TA (%)					
AF	0.4 ± 0.3 ^aA^	0.4 ± 0.3 ^aA^	0.4 ± 0.4 ^aA^	0.4 ± 0.4 ^aA^	0.4 ± 0.4 ^aA^	0.4 ^a^
P	0.4 ± 0.4 ^aA^	0.4 ± 0.3 ^aA^	0.4 ± 0.3 ^aA^	0.4 ± 0.3 ^aA^	0.4 ± 0.3 ^aA^	0.4 ^a^
Mean	0.4 ^A^	0.4 ^A^	0.40 ^A^	0.4 ^A^	0.4 ^A^	
	SS/TA					
AF	11.1 ± 0.3 ^aA^	11.8 ± 0.2 ^aA^	12.4 ± 0.4 ^aA^	11.8 ± 0.4 ^aA^	12.2 ± 0.4 ^aA^	11.9 ^a^
P	10.6 ± 0.4 ^aA^	11.5 ± 0.3 ^aA^	11.2 ± 0.3 ^aA^	11.9 ± 0.3 ^aA^	11.5 ± 0.3 ^aA^	11.4 ^a^
Mean	10.9 ^A^	11.6 ^A^	11.8 ^A^	11.9 ^A^	11.9 ^A^	
	Lycopene (mg 100 g^−1^)					
AF	0.3 ± 0.2 ^bA^	0.4 ± 0.4 ^bA^	0.9 ± 0.3 ^bB^	0.1 ± 0.0 ^aA^	0.7 ± 0.3 ^aA^	0.5 ^b^
P	0.5 ± 0.3 ^aA^	0.6 ± 0.2 ^aA^	1.9 ± 0.2 ^aA^	0.1 ± 0.0 ^bA^	0.7 ± 0.1 ^aA^	0.8 ^a^
Mean	0.4 ^BC^	0.5 ^B^	1.4 ^A^	0.1 ^C^	0.7 ^B^	
	β-carotene (mg 100 g^−1^)					
AF	0.4 ± 0.3 ^bA^	0.5 ± 0.3 ^bA^	0.4 ± 0.1 ^bB^	0.2 ± 0.0 ^bA^	0.4 ± 0.2 ^bA^	0.4 ^b^
P	0.8 ± 0.2 ^aA^	0.6 ± 0.3 ^aA^	1.3 ± 0.1 ^aA^	0.3 ± 0.0 ^aA^	0.6 ± 0.4 ^aA^	0.7 ^a^
Mean	0.6 ^AB^	0.5 ^BC^	0.9 ^A^	0.2 ^C^	0.5 ^BC^	

* Values are presented as mean ± standard error. Means followed by the same lowercase letters within columns (comparison between protected environment conditions (PECs)) and uppercase letters within rows (comparison among tomato hybrids (THs)) are not significantly different according to Tukey’s test (*p* ≤ 0.05).

## Data Availability

The data used and/or analyzed in this study are available from the corresponding author on reasonable request.

## Supplementary Materials

The following supporting information can be downloaded at: https://www.mdpi.com/article/10.3390/metabo16060389/s1, Figure S1: Schematic representation of a greenhouse covered with twin-walled polycarbonate panels equipped with a laminar water flow cooling system.

## Abbreviations

The following abbreviations are used in this manuscript:

AF	agricultural film
P	polycarbonate panels
PAR	photosynthetically active radiation
TA	titratable acidity
TSS	total soluble solids
ANOVA	analysis of variance
PCA	principal component analysis
SVD	singular value decomposition
PSII	photosystem II
ROS	reactive oxygen species
VPD	vapor pressure deficit
PEC	protected environment conditions
TH	tomato hybrids
CV	coefficient of variation
DB	dry biomass
LA	leaf area
PL	plant length
SDB	shoot dry biomass
Y	yield
MY	marketable yield
NF	number of fruits
FW	fruit fresh weight
FL	fruit length
FD	fruit diameter
C*	chroma
h°	hue angle
SS/TA	soluble solids to titratable acidity ratio
βc	β-carotene
LY	lycopene
RI	ripening index

## References

[1] Bita C.E., Gerats T. (2013). Plant tolerance to high temperature in a changing environment: Scientific fundamentals and production of heat stress-tolerant crops. Front. Plant Sci..

[2] Camejo D., Jiménez A., Alarcón J.J., Torres W., Gómez J.M., Sevilla F. (2006). Changes in photosynthetic parameters and antioxidant activities following heat-shock treatment in tomato plants. Funct. Plant Biol..

[3] Khan Q., Wang Y., Xia G., Yang H., Luo Z., Zhang Y. (2024). Deleterious effects of heat stress on the tomato, its innate responses, and potential preventive strategies in the realm of emerging technologies. Metabolites.

[4] Kim J., Jeong H., Kim S., Chae W. (2025). Pollen traits significantly associated with fruit yield traits under heat stress among large fruit but not cherry fruit tomatoes. Hortic. Environ. Biotechnol..

[5] Oliveira F.F.C., Teixeira A.S., Sousa A.B.O., Liberato G.A., Lima G.S., Rosal G.B., Rocha Neto O. (2025). Plastic coverings on the environment of agricultural greenhouses. Rev. Bras. Eng. Agríc. Ambient..

[6] Pataraico Junior V., Fernandes Junior F., Zanuzo M.R., Silva Campos R.A., Silva Ponce F., Botelho S.D.C.C., Silva V., Antunes D.T., Nascimento M.S.P., Seabra Junior S. (2023). Yield performance and nutritional quality of tomato hybrids in response to protected environments during the Amazonian summer. Adv. Hortic. Sci..

[7] Chimankare R.V., Das S., Kaur K., Magare D. (2023). A review study on the design and control of optimised greenhouse environments. J. Trop. Ecol..

[8] Yusuf A.G., Al-Yahya F.A., Saleh A.A., Abdel-Ghany A.M. (2025). Optimizing greenhouse microclimate for plant pathology: Challenges and cooling solutions for pathogen control in arid regions. Front. Plant Sci..

[9] Yeshiwas Y., Tadele E., Mohammed A., Hu X. (2026). Impacts of climate change on horticultural systems with focus on socio-economic implications, production and postharvest challenges, and adaptive pathways. Discov. Environ..

[10] Maraveas C., Kotzabasaki M.I., Bayer I.S., Bartzanas T. (2023). Sustainable greenhouse covering materials with nano- and micro-particle additives for enhanced radiometric and thermal properties and performance. AgriEngineering.

[11] Maraveas C. (2023). Sustainable greenhouse covering materials with nano- and micro-structures. Nanomaterials.

[12] Holcman E., Sentelhas P.C., Mello S.D.C. (2015). Microclimatic changes caused by different plastic coverings in greenhouses cultivated with cherry tomato in southern Brazil. Rev. Bras. Meteorol..

[13] Santos J.C.P., Temp M.S., Fernandes M.R., Lima R.C.A. (2018). Comportamento ótico de vidros e policarbonatos translúcidos frente à radiação solar. Matéria.

[14] Kim H.K., Lee S.Y., Kim J.K. (2022). Evaluating the effect of cover materials on greenhouse microclimates and thermal performance. Agronomy.

[15] Antunes D.T., Silva Ponce F., Pataraico Junior V., Fernandes Junior F., Silva Campos R.A., Botelho S.D.C.C., Nascimento M.S.P., Seabra Júnior S., Zanuzo M.R. (2022). Effect of polycarbonate and agricultural film on production and biochemical compounds of tomato fruits. Res. Soc. Dev..

[16] Mohammed M.A., Menkabo M.M., Budaiwi I.M. (2023). Assessment of polycarbonate material as a sustainable substitute for glazing in hot climates. Int. J. Sustain. Energy.

[17] McCartney L., Lefsrud M. (2018). Protected agriculture in extreme environments: A review of controlled environment agriculture in tropical, arid, polar, and urban locations. Appl. Eng. Agric..

[18] Korkmaz C., Tezcan N.Y. (2025). The temporal variation of some physical and mechanical properties of different polycarbonate greenhouse covering materials and the detection of aging using thermal imaging. Infrared Phys. Technol..

[19] Menkabo M.M., Alhaji M.M., Budaiwi I.M., Abdou A.A. (2024). Thermo-environmental performance of polycarbonate materials as a glazing substitute in hot climates. Adv. Build. Energy Res..

[20] Dos Santos T.B., Ribas A.F., Souza S.G.H., Budzinski I.G.F., Domingues D.S. (2022). Physiological responses to drought, salinity, and heat stress in plants: A review. Stresses.

[21] Francesca S., Vitale L., Arena C., Raimondi G., Olivieri F., Cirillo V., Paradiso A., de Pinto M.C., Maggio A., Barone A. (2022). The efficient physiological strategy of a novel tomato genotype to adapt to chronic combined water and heat stress. Plant Biol..

[22] Visakh R.L., Anand S., Reddy N.R.S., Jha U.C., Sah R.P., Beena R. (2026). Scientific insights into genetic and physiological response of heat stress in tomato. Planta.

[23] Fernández-Crespo E., Liu-Xu L., Albert-Sidro C., Scalschi L., Llorens E., González-Hernández A.I., Crespo O., Gonzalez-Bosch C., Camañes G., García-Agustín P. (2022). Exploiting tomato genotypes to understand heat stress tolerance. Plants.

[24] Graves M., Graves T., Yuan B., Leng P. (2026). ABA-mediated reprogramming of carotenoid metabolism under heat stress impairs tomato fruit ripening. Plant Sci..

[25] Stoleru V., Inculet S.C., Mihalache G., Cojocaru A., Teliban G.C., Caruso G. (2020). Yield and nutritional response of greenhouse grown tomato cultivars to sustainable fertilization and irrigation management. Plants.

[26] Gong X., Li X., Qiu R., Bo G., Ping Y., Xin Q., Ge J. (2022). Ventilation and irrigation management strategy for tomato cultivated in greenhouses. Agric. Water Manag..

[27] Furlani P.R., Silveira L.C.P., Bolonhezi D., Faquin V. (1999). Cultivo Hidropônico de Plantas.

[28] Nagata M., Yamashita I. (1992). Simple method for simultaneous determination of chlorophyll and carotenoids in tomato fruit. Nippon Shokuhin Kogyo Gakkaishi.

[29] Zamani F., Duri L.G., Mori M., Paradiso R. (2025). Advances in light manipulation in greenhouse horticulture: The innovative smart covers. Front. Plant Sci..

[30] Mogharreb M.M., Abbaspour-Fard M.H. (2019). Experimental study on the effect of a novel water injected polycarbonate shading on light transmittance and greenhouse interior conditions. Energy Sustain. Dev..

[31] Murata N., Takahashi S., Nishiyama Y., Allakhverdiev S.I. (2007). Photoinhibition of photosystem II under environmental stress. Biochim. Biophys. Acta Bioenerg..

[32] Gururani M.A., Venkatesh J., Tran L.S.P. (2015). Regulation of photosynthesis during abiotic stress-induced photoinhibition. Mol. Plant.

[33] Allakhverdiev S.I., Kreslavski V.D., Klimov V.V., Los D.A., Carpentier R., Mohanty P. (2008). Heat stress: An overview of molecular responses in photosynthesis. Photosynth. Res..

[34] Lu T., Meng Z., Zhang G., Qi M., Sun Z., Liu Y., Li T. (2017). Sub-high temperature and high light intensity induced irreversible inhibition on photosynthesis system of tomato plant (*Solanum lycopersicum* L.). Front. Plant Sci..

[35] Kübarsepp L., Laanisto L., Niinemets Ü., Talts E., Tosens T. (2020). Are stomata in ferns and allies sluggish? Stomatal responses to CO_2_, humidity and light and their scaling with size and density. New Phytol..

[36] Rashid M.A., Andersen M.N., Wollenweber B., Kørup K., Zhang X., Olesen J.E. (2018). Impact of heat-wave at high and low VPD on photosynthetic components of wheat and their recovery. Environ. Exp. Bot..

[37] Rashid M.A., Andersen M.N., Wollenweber B., Zhang X., Olesen J.E. (2018). Acclimation to higher VPD and temperature minimized negative effects on assimilation and grain yield of wheat. Agric. For. Meteorol..

[38] Grossiord C., Buckley T.N., Cernusak L.A., Novick K.A., Poulter B., Siegwolf R.T., McDowell N.G. (2020). Plant responses to rising vapor pressure deficit. New Phytol..

[39] Shi D., Huang Q., Liu Z., Liu T., Su Z., Guo S., Yang X. (2022). Radiation use efficiency and biomass production of maize under optimal growth conditions in Northeast China. Sci. Total Environ..

[40] Jiang C., Johkan M., Hohjo M., Tsukagoshi S., Ebihara M., Nakaminami A., Maruo T. (2017). Photosynthesis, plant growth, and fruit production of single-truss tomato improves with supplemental lighting provided from underneath or within the inner canopy. Sci. Hortic..

[41] Heuvelink E. (2018). Tomatoes: Crop Production Science in Horticulture.

[42] Gregoriou K., Pontikis K., Vemmos S. (2007). Effects of reduced irradiance on leaf morphology, photosynthetic capacity, and fruit yield in olive (*Olea europaea* L.). Photosynthetica.

[43] Poorter H., Niinemets Ü., Ntagkas N., Siebenkäs A., Mäenpää M., Matsubara S., Pons T. (2019). A meta-analysis of plant responses to light intensity for 70 traits ranging from molecules to whole plant performance. New Phytol..

[44] Panda D., Mohanty S., Das S., Mishra B., Banerjee S., Kumar A., Behera L. (2025). Shade tolerance is associated with foliar adaptations, improved radiation use efficiency, and photosynthetic rate in rice. Sci. Rep..

[45] O’Carrigan A., Hinde E., Lu N., Xu X.Q., Duan H., Huang G., Chen Z.H. (2014). Effects of light irradiance on stomatal regulation and growth of tomato. Environ. Exp. Bot..

[46] Moreno-Teruel M.A., Molina-Aiz F.D., Peña-Fernández A., López-Martínez A., Valera-Martínez D.L. (2021). The effect of diffuse film covers on microclimate and growth and production of tomato (*Solanum lycopersicum* L.) in a Mediterranean greenhouse. Agronomy.

[47] Liu H., Shao M., Yang L. (2023). Photosynthesis characteristics of tomato plants and its responses to microclimate in new solar greenhouse in North China. Horticulturae.

[48] Thakur N., Sharma D., Kaur J., Sharma V. (2025). Characterizing tomato genotypes in the varied climates of north-western Himalayas and implications for environmental resilience using GGE biplot analyses. Sci. Rep..

[49] Oliva-Ruiz M., Pacheco N., Cuevas-Bernardino J.C., Tezara W., De-la-Peña C., Andueza-Noh R.H., Garruña R. (2023). Growth, phenotypic plasticity and fruit quality in tomato: A study under high temperature and elevated CO_2_. Horticulturae.

[50] Silva A.G., Costa E., Zoz T., Binotti F.D.S. (2021). Micrometeorological characterization of protected environments for plant production. Rev. Agric. Neotrop..

[51] Francesca S., Vitale L., Graci S., Addonizio M., Barone A., Rigano M.M. (2024). Integrated physiological and genetic data reveal key traits for heat tolerance in tomato. Plant Stress.

[52] Lou H., Li S., Shi Z., Zou Y., Zhang Y., Huang X., Xu C. (2025). Engineering source-sink relations by prime editing confers heat-stress resilience in tomato and rice. Cell.

[53] Borguini R.G., Silva M.V. (2005). Características físico-químicas e sensoriais do tomate (*Lycopersicon esculentum*) produzido por cultivo orgânico em comparação ao convencional. Alim. Nutr..

[54] Vitale E., Velikova V., Tsonev T., Costanzo G., Paradiso R., Arena C. (2022). Manipulation of light quality is an effective tool to regulate photosynthetic capacity and fruit antioxidant properties of *Solanum lycopersicum* L. cv. ‘Microtom’ in a controlled environment. PeerJ.

[55] Murariu O.C., Brezeanu C., Jităreanu C.D., Robu T., Irimia L.M., Trofin A.E., Brezeanu P.M. (2021). Functional quality of improved tomato genotypes grown in open field and in plastic tunnel under organic farming. Agriculture.

[56] Singh D.P., Rai N., Farag M.A., Maurya S., Yerasu S.R., Bisen M.S., Behera T.K. (2024). Metabolic diversity, biosynthetic pathways, and metabolite biomarkers analysed via untargeted metabolomics and the antioxidant potential reveal for high temperature tolerance in tomato hybrid. Plant Stress.

[57] Seabra Junior S., Casagrande J.G., Toledo C.A., Ponce F.S., Ferreira F.S., Zanuzo M.R., Lima G.P.P. (2022). Selection of thermotolerant Italian tomato cultivars with high fruit yield and nutritional quality for the consumer taste grown under protected cultivation. Sci. Hortic..

[58] Zhao Z., Liu Z., Mao X. (2020). Biotechnological advances in lycopene β-cyclases. J. Agric. Food Chem..

[59] Wang Y., Zhang C., Xu B., Fu J., Du Y., Fang Q., Zhao H. (2022). Temperature regulation of carotenoid accumulation in the petals of sweet osmanthus via modulating expression of carotenoid biosynthesis and degradation genes. BMC Genom..

[60] Havaux M. (2014). Carotenoid oxidation products as stress signals in plants. Plant J..

[61] Vardanian I., Sargsyan G., Martirosayn G., Pahlevanyan A., Tsereteli I., Martorosyan H., Harutyunyan Z. (2025). Lycopene in tomatoes: Genetic regulation, agronomic practices, and environmental influence. Funct. Food Sci..

[62] Wang J., Wu Y., Yu J. (2026). The carotenoids metabolism during tomato fruit ripening: Insights into multi-level regulatory network. BMC Plant Biol..

[63] Amr A., Raie W. (2022). Tomato components and quality parameters. A review. Jordan J. Agric. Sci..

[64] Kader A.A. (2002). Quality parameters of fresh-cut fruit and vegetable products. Fresh-Cut Fruits and Vegetables.

[65] Dorais M., Ehret D.L., Papadopoulos A.P. (2008). Tomato (*Solanum lycopersicum*) health components: From the seed to the consumer. Phytochem. Rev..

[66] Trento A., Antunes T., Fernandes Junior F., Zanuzo M., Dallacort R., Seabra Junior S. (2021). Desempenho de cultivares de tomate italiano de crescimento determinado em cultivo protegido sob altas temperaturas. Nativa.

