# Supplementary material for: Metabolism and Yield of Grape–Tomato Hybrids Under Heat Stress in an Innovative Protected Environment Using Twin-Walled Polycarbonates with Laminar Water Flow
Source: Metabolites. 2026 Jun 4;16(6):389. doi: 10.3390/metabo16060389 (PMC13303930; doi:10.3390/metabo16060389)
Supplement: Supplementary file 1 [file metabolites-16-00389-s001.zip › Figure S1.pdf]

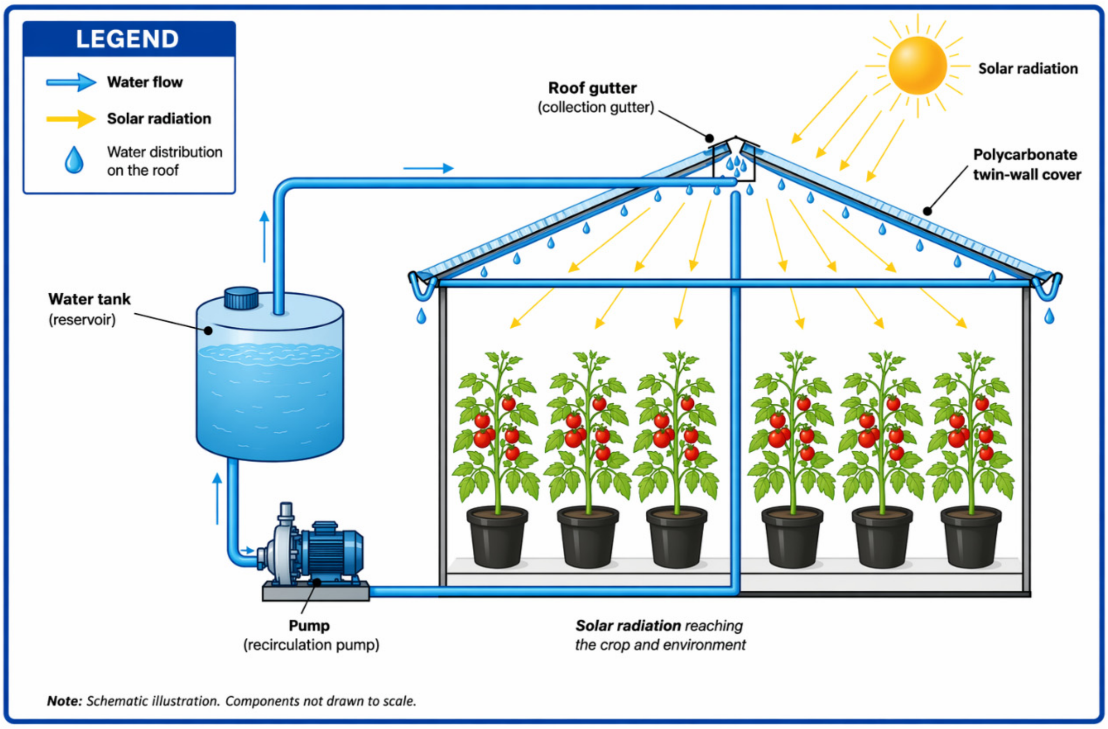

Figure S1. Schematic representation of a greenhouse covered with polycarbonate twin-wall panels equipped with a laminar water flow cooling system.
